# Innumerable Culture-Negative Intracranial Abscesses in an Immunocompetent Young Male Diagnosed by 16s rRNA Gene Sequencing

**DOI:** 10.7759/cureus.71475

**Published:** 2024-10-14

**Authors:** Ronald A Miller, Yeonsoo Kim, Vasyl Pryshliak, Timothy M Beutler

**Affiliations:** 1 Neurology, State University of New York (SUNY) Upstate Medical University, Syracuse, USA; 2 Neurosurgery, State University of New York (SUNY) Upstate Medical University, Syracuse, USA; 3 Pathology, State University of New York (SUNY) Upstate Medical University, Syracuse, USA

**Keywords:** 16s rrna gene sequencing analysis, brain abscesses, intraparenchymal abscess, streptococcus anginosus group, streptococcus intermedius bacteremia

## Abstract

Here, we present a case of a healthy young man with an initial presentation concerning for infectious encephalitis later found on neuroimaging to have extensive intracranial lesions. Pathological analysis of these lesions was consistent with a bacterial intraparenchymal abscess; however, cultures were negative for a causative organism. Results of gene sequencing from an abscess sample were consistent with *Streptococcus intermedius* infection. *Streptococcus intermedius*, a β-hemolytic gram-positive species of the *Streptococcus anginosus* group (SAG), is known to possess several virulence factors that promote abscess formation.

## Introduction

This case demonstrates that even in an immunocompetent host, intraparenchymal abscesses can be numerous and broadly distributed within the central nervous system (CNS). Despite mild clinical signs on initial presentation, the lesion burden was impressive, extending bilaterally to supra- and infratentorial regions. Further, the challenge of isolating the causal pathogen was an additional hurdle, as culture results collected from intracranial samples were uninformative. This case highlights the importance of culture-independent means of pathogen detection.

## Case presentation

**Figure 1:**
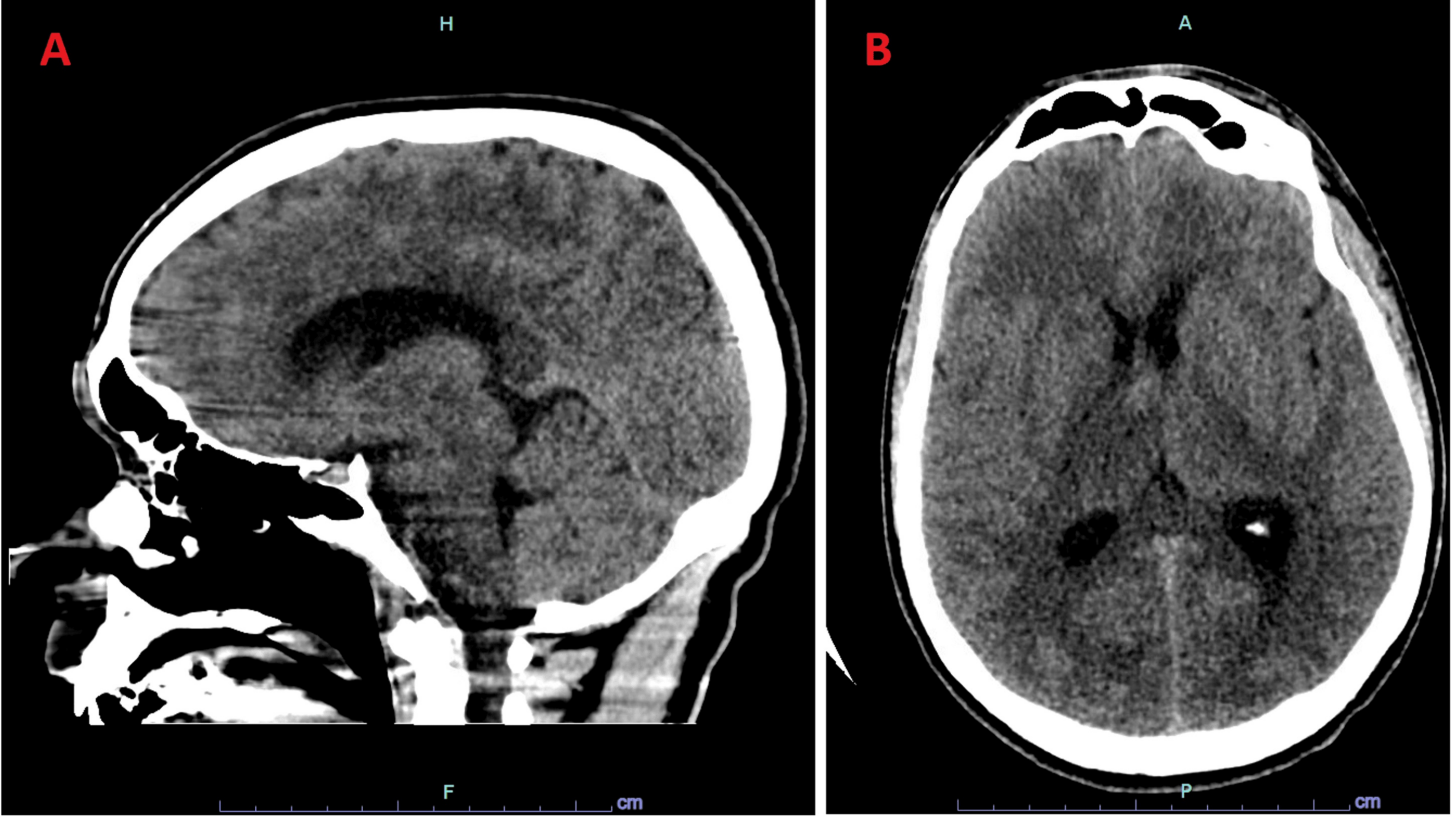
Sagittal and axial non-contrast CT of the head at presentation. Sagittal (A) and axial (B) views of the initial CT imaging which was negative for acute hemorrhage but demonstrated questionable patchy decreased attenuation in the bilateral parietal lobes and right thalamus. CT: computed tomography

A 26-year-old man presented with a five-day history of progressive confusion, somnolence, nausea, vomiting, headache, and fever. He sustained a temperature of 38.9, a leukocytosis of 19.8 (leukocytes/L), and a positive COVID-19 respiratory panel. Initial non-contrast computed tomography (CT) of the head demonstrated no acute intracranial pathology (Figure 1). The patient was pan-cultured and placed on ceftriaxone, vancomycin, acyclovir, and remdesivir. Remdesivir was started due to the detection of polymerase chain reaction (PCR)-positive COVID-19 at the time of presentation.

**Figure 2:**
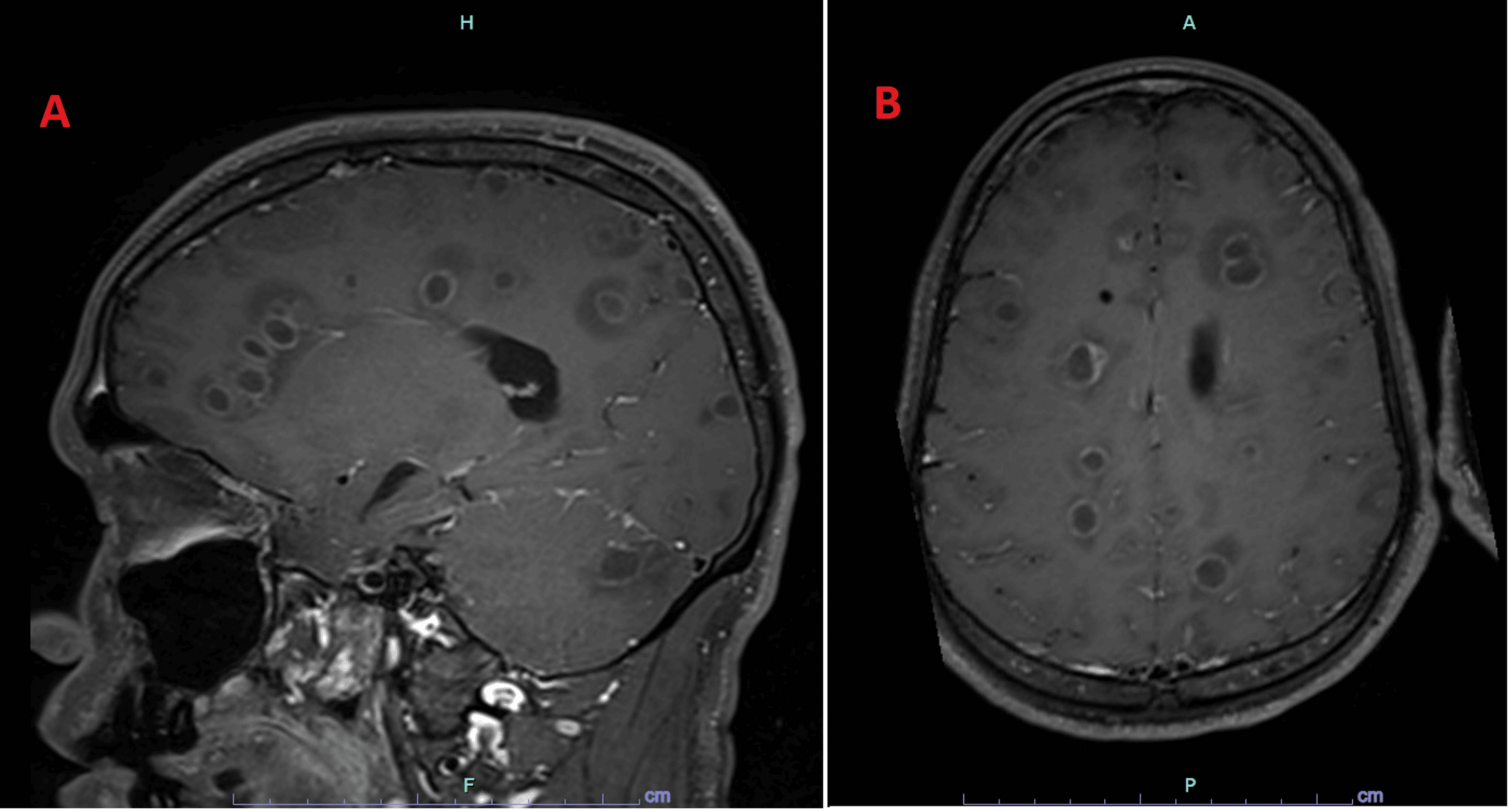
MRI of multifocal bilaterally disseminated lesions. Sagittal (A) and axial (B) MRI demonstrating bilateral lesions disseminated around the lateral ventricles and throughout the supra- and infratentorial regions (T1 3D space G+ MPR). MRI: magnetic resonance imaging; MPR: multiplanar reformation or reconstruction

Over several hours, the patient's neurological exam deteriorated. Ultimately, he was unable to follow commands and exhibited urinary incontinence and decorticate posturing. The patient desaturated to a SpO2 of 80% on 15 L oxygen and was intubated for airway protection. A rapid CT demonstrated ventriculomegaly and an external ventricular drain (EVD) was emergently placed. Magnetic resonance imaging (MRI) (Figure 2) revealed innumerable rim-enhancing lesions scattered bilaterally throughout the cerebral and cerebellar hemispheres and brainstem. While in the intensive care unit (ICU), the patient required mechanical ventilation, intravenous (IV) sedation with dexmedetomidine and fentanyl, and intracranial pressure (ICP) monitoring through the EVD. 

The patient had no significant past medical history or recent travel, was heterosexual with one sexual partner, had no IV drug use, ate meat (including pork), and was negative for human immunodeficiency virus (HIV) and syphilis. Cerebrospinal fluid (CSF), collected after antibiotic administration, was notable for RBC 904, mild lymphocytic pleocytosis (49 nucleated cells with 43% lymphocytes), and a negative pathogen panel (including negative for cryptococcal antigen) (see Table 1 for the full CSF chemistry). Echinococcus and coccidioidomycosis serology and toxoplasma IgG were negative. Fundus exam and transthoracic echocardiogram (TTE) were unremarkable.

**Table 1:** CSF chemistry. CSF: cerebrospinal fluid; RBC: red blood cell

CSF	
Clarity	Hazy
RBC count	904
Total nucleated cells	49
Color	Colorless
Glucose	84
Lymphocyte	43
Monocyte/macrophage	25
Neutrophil	32
Protein	17

**Figure 3:**
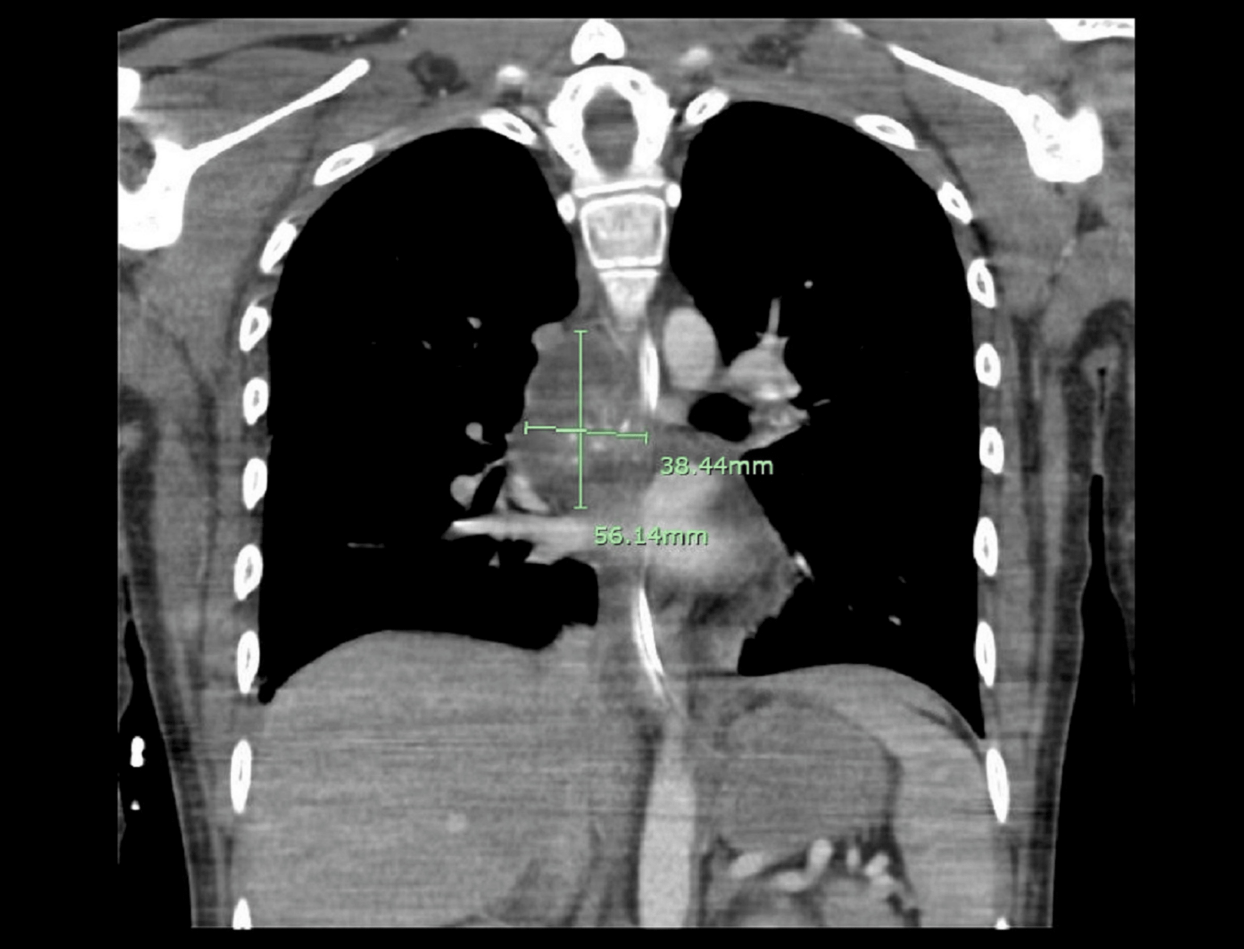
CT of the thorax with contrast. Coronal CT of the thorax with contrast demonstrating a partially calcified posterior mediastinal lesion measuring 4.7×2.6×4.7 cm. CT: computed tomography

**Figure 4:**
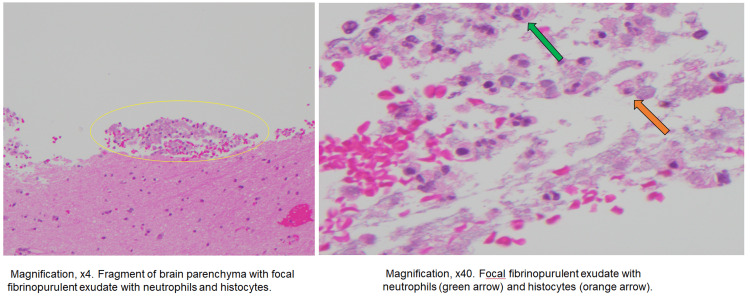
Micropathology of the surgical biopsy. Pathology of the brain parenchyma with focal fibropurulent exudate at 4× (left) and 40× (right) magnification. Neutrophils and histiocytes are indicated on the right.

CT of the thorax, ordered for further investigation of possible malignancy, demonstrated a 4.7×2.6×4.7 cm partially calcified posterior mediastinal mass (Figure 3). Fine needle aspiration (FNA) of the subcarinal lymph node yielded a purulent material, and an anaerobic culture from the aspirate grew *Streptococcus intermedius* (*Streptococcus anginosus* group). Brain biopsy collected from the intracranial lesions displayed microscopic findings consistent with abscesses showing many neutrophils and foamy histiocytes (Figure 4). However, Gram stain, Grocott methenamine silver (GMS) stain, acid-fast bacillus (AFB) (Ziehl-Neelsen), and immunohistochemical stain targeting *Toxoplasma* were all negative.

**Figure 5:**
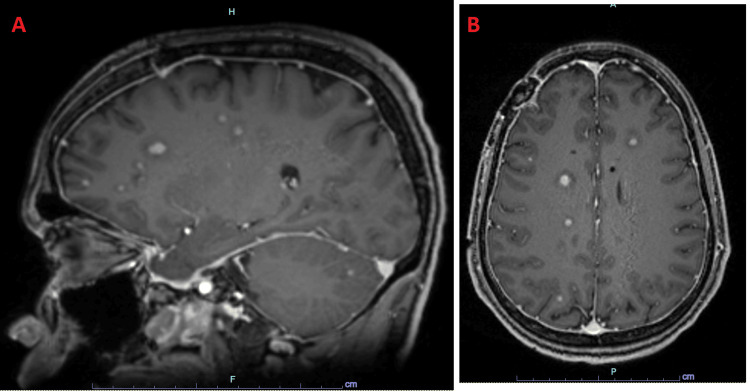
Sagittal and axial MRI three months after presentation. Sagittal (A) and axial (B) MRI three months following the initial presentation demonstrating a marked interval decrease in lesion sizes as well as a reduction in edema (T1 3D space G+ MPR). MRI: magnetic resonance imaging; MPR: multiplanar reformation or reconstruction

The patient later revealed a dental implant was placed two weeks prior to symptom onset. Although the lymph node culture was strongly suggestive of an odontogenic source of infection, the lack of any culture growth from the intracranial sample left a degree of ambiguity to the source of the CNS lesions. After several weeks of treatment on ceftriaxone and metronidazole without significant improvement on the exam, a brain biopsy sample (taken from the original collection) was sent to the University of Washington Molecular Diagnostics Laboratory (UW-MDL) for 16S rRNA gene sequencing. PCR results were positive for *Streptococcus intermedius* and *Pichia jadinii*, the latter being a rare yeast organism with low virulence thought to be a contaminant. These results increased our confidence that the lymph node biopsy was reflective of the responsible pathogen and the negative results of the brain biopsy culture were caused by antibiotic suppression, as it was obtained after several days on empiric antibiotic therapy. The PCR results also precluded the need for further intracranial biopsy or unnecessary escalation of antibiotic therapy. The patient's antibiotic therapy was further narrowed to IV ceftriaxone for an additional four more weeks. Follow-up MRI (Figure 5) showed a marked reduction in the size of intracranial abscesses. After one month in the ICU, the patient completed an additional three weeks of intensive rehabilitation.

At the time of his follow-up visit, two months after his initial presentation, he was functioning independently. His neurological exam demonstrated normal mental status, no deficits in language comprehension or speech production, and no cranial nerve deficits. Also, his strength, sensation, and reflexes were normal in all extremities. 

## Discussion

Brain abscesses represent a relatively minor portion of CNS infections, between 1% and 2% in developed nations [1]. However, the high morbidity and mortality associated with an intraparenchymal abscess make early recognition vital. Clinical signs are a function of the extent of inflammation (cerebral edema) and lesion location. The classical triad of headache, fever, and focal neurologic deficits is present in only 20% of cases [2]. The pathogenic mechanism of an intraparenchymal abscess is largely dependent on preexisting conditions, especially comorbidities affecting immune function, with bacteria representing more than 95% of abscesses in immunocompetent patients [3].

Microbes causing brain abscesses may originate from various sources, including hematogenous spread, neurosurgical procedures, head trauma, or contiguous spread [4]. Hematogenous spread is most common, often linked to endocarditis, pulmonary infections, cyanotic heart disease, and pulmonary arteriovenous malformation. Odontogenic infections have been identified as a source of bacterial brain abscesses, particularly through cavernous sinus, hematogenous, or lymphatic routes, especially in immunocompromised patients [5]. Notably, a case study revealed brain abscesses in a hereditary hemorrhagic telangiectasia patient due to the metastatic spread of methicillin-resistant *Staphylococcus aureus*, *Streptococcus intermedius*, and *Candida guilliermondii* from a pulmonary arteriovenous malformation [6]. Such an example underscores that while many brain abscesses stem from localized infections like odontogenic or nasopharyngeal sources, metastatic conditions can also contribute [6].

The current case was noteworthy for the large number and broad dissemination of intracranial abscesses on presentation despite the patient's immunocompetent status. *Streptococcus intermedius* is a gram-positive coccus bacterium that is associated with ring-enhancing brain lesions and is a part of the *Streptococcus anginosus* group (SAG), also known as *Streptococcus milleri* [7,8]. Prior work has shown that several virulence factors of *Streptococcus intermedius*, including a polysaccharide capsule and adherence to extracellular matrix (ECM) proteins, lead to longer hospital stays and higher mortality rates [9]. An analysis of over 100 cases of *Streptococcus intermedius* reported that most patients were older aged 61-70 years old; however, the range was anywhere between five and 83 years with the most common symptoms presenting as intermittent fever and headaches [10]. Several common risk factors including previous dental procedures or sinusitis followed by diabetes mellitus have been shown to increase *Streptococcus intermedius* infections [10]. *Streptococcus* spp. are among the most commonly cultured organisms of brain abscesses from a dental source [4]. Immunocompromised individuals or patients with comorbidities are affected the most by these opportunistic bacteria SAG, which are part of the normal flora of the digestive, reproductive, and most importantly respiratory tract [11].

A recently published case of brain abscesses caused by *Streptococcus intermedius* reported striking similarities to the current presentation [12]. In both cases, a young healthy, immunocompetent male presented with several intracranial lesions in addition to mediastinal lymph node involvement. In each instance, the gram stain from intracranial abscess sites was culture-negative, perhaps due to the early application of empiric antibiotics. However, 16S rRNA sequencing proved to be instrumental in isolating the presence of the *Streptococcus *species in the brain. This enabled greater confidence in narrowing the antibiotic therapy to target a monomicrobial infection.

Culture negativity from collected intracranial abscesses is common and strongly depends on the relationship between the onset of therapy and the time of sample collection. Studies estimate that anywhere from 25% to 40% of samples are culture-negative [13,14]. Pyrosequencing, or the use of gene amplification methods to detect the genetic signatures of pathogens, has been shown to be significantly more sensitive, often revealing the polymicrobial nature of infections, in detecting etiologic organisms from abscess samples when compared to culture-based methods [15-17]. Ideally, these gene sequencing methods will become routine, obviating the need for sending out samples to national laboratory centers which can delay care and narrowing of antimicrobial therapy.

## Conclusions

This case suggests that *Streptococcus intermedius *is a disease entity that should be strongly considered on the differential for an immunocompetent patient with disseminated intracranial culture-negative bacterial abscesses, and genetic testing should be completed early in the disease course to expedite appropriate treatment. Fortunately, broad-spectrum antibiotic therapy, a standard initial treatment regimen, remains effective against this commonly isolated pathogen.
